# Glutamate dehydrogenase 2 is required for virulence by facilitating fungal growth in the host hemocoel

**DOI:** 10.1080/21505594.2025.2591402

**Published:** 2025-11-26

**Authors:** Yuzhen Lu, Denghui Wu, Jiawei Hu, Guojun Peng, Jielai Zhong, Yan Liu, Jing Li, Qiang Gao, Xiao-Qiang Yu

**Affiliations:** a Guangdong Provincial Key Laboratory of Insect Developmental Biology and Applied Technology, Guangzhou Key Laboratory of Insect Development Regulation and Application Research, Institute of Insect Science and Technology, School of Life Sciences, South China Normal University, Guangzhou, China; b School of Biomedical Sciences, Hunan University, Changsha, Hunan, China

**Keywords:** Glutamate dehydrogenase 2, virulence, *M. robertsii*, ammonia, fungal colonization

## Abstract

*Metarhizium robertsii*, a fungal pathogen employed in pest biocontrol, can alkalize the surrounding environment, although the biological implications remain unclear. Here, we found that *M. robertsii* glutamate dehydrogenase 2 (*MrGDH2*) was responsible for alkalization during fungal growth on insect wings or media containing cuticle powder. Loss of *MrGDH2* function resulted in significantly reduced virulence during both cuticle-passing and cuticle-bypassing infections but did not affect appressorium formation or cuticle penetration. Deletion of *MrGDH2* failed to alkalize amino acid-containing media under carbon deprivation, leading to impaired mycelia growth and conidiation. Hemolymph carbohydrates were decreased during *M. robertsii* infection, and the Δ*Mrgdh2* mutant exhibited delayed fungal growth and impaired alkalization in hemolymph cultures. Notably, expression of *PacC*, a pH-responsive transcription factor critical for virulence, was downregulated in hyphal bodies of the Δ*Mrgdh2* mutant. Contrary to the established models in plant and human fungal pathogens, we demonstrate that Gdh2 activity is dispensable for appressorium formation but essential for fungal colonization in insect hemocoel during *M. robertsii* infection.

## GRAPHICAL ABSTRACT

**Figure:**
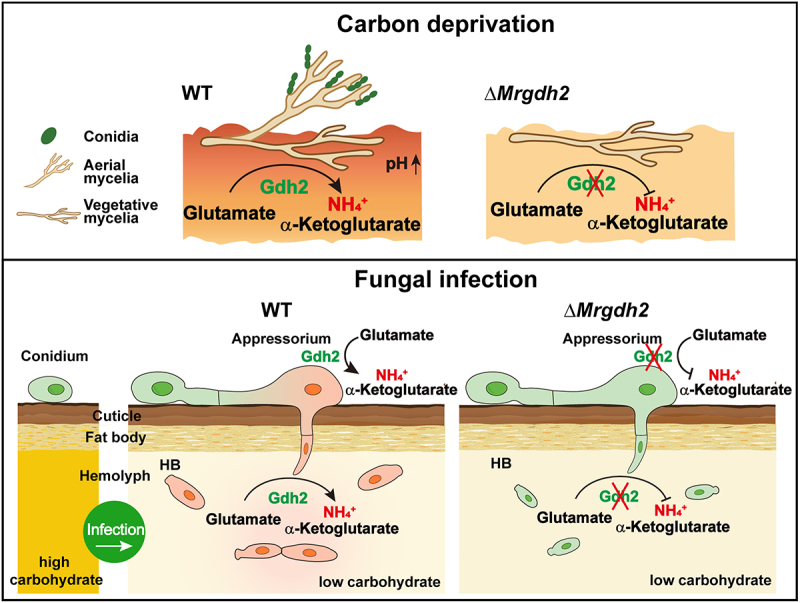

## Introduction

Environmental pH, one of the most crucial physiochemical variables, influences not only cell growth and development but also the pathogen’s ability to colonize, penetrate, and eventually kill the target host [1]. Phytopathogenic fungi are able to modify environmental pH, and the accumulation of ammonium has been linked to the pathogenicity in *Colletotrichum* species, such as *C. coccodes* and *C. gloeosporioides* [2]. This ambient alkalization mechanism is accomplished by the active release of ammonia or small regulatory peptides from fungi that mimic plant rapid alkalinizing factors (RALFs) [3,4].

The primary source of free ammonia in *Saccharomyces cerevisiae* is generated by the mitochondron-localized NAD^+^-dependent glutamate dehydrogenase (Gdh2), which catalyzes the deamination of glutamate to α-ketoglutarate, resulting in the production of NADH and NH_3_ [5]. Via α-ketoglutarate, an important intermediate for many biological processes, Gdh2 connects amino acid metabolism to the tricarboxylic acid cycle (TCA cycle) [6]. Deletion of *GHD2* resulted in reduced generation of ammonia in *C. gloeosporioide* and also 85% suppression of appressorium formation, as well as a decrease in pathogenicity [7]. Exogenous addition of ammonia could fix the deficiency in appressoria formation [7]. *Mgd1* (*GDH2* homolog) was highly expressed in *Magnaporthe oryzae* appressoria, and deleting *Mgd1* decreased the ability to form the appressoria and resulted in a weaker virulence [8]. *Candida albicans* strains lacking *GDH2* (*gdh2*^−/−^) did not extrude ammonia on amino acid-based media, but *gdh2*^−/−^ mutants were as virulent as the wild-type strain [9].

Entomopathogenic fungi, such as *Metarhizium robertsii*, are used as biological control agents against a wide range of agricultural pests [10]. The conidia that germinate on an insect’s cuticle develop into an infectious structure known as an appressorium. Then the appressorium penetrates the cuticle via a synergistic action of turgor pressure and cuticle-degrading enzymes [11]. When the fungal hyphae successfully invade insect hemolymph, they transform into yeast-like cells known as blastospores or hyphal bodies. After insects are killed, the fungus in the hemolymph re-breaches the insect cuticle and produces multilayered spores that cover the cadaver’s surface [10].

Previous studies showed that *M. robertsii* can generate ammonia to alkalize the media and increase the pH of insect cuticle during infection [12,13]. However, the role of ambient alkalinization during *M. robertsii* infection remains undefined. In this study, we showed that *M. robertsii* Gdh2 (MrGdh2) was responsible for alkalization by catalyzing glutamate deamination and was required for amino acid-dependent fungal growth and reproduction during carbon deprivation. In contrast to plant fungal pathogens, MrGdh2 activity was not required for appressorium formation and cuticle penetration in *M. robertsii*. Differing from the human fungal pathogen *C. albicans*, MrGdh2 was essential for virulence by facilitating colonization in insect hemocoel. We therefore uncovered the unique role of Ggh2-dependent alkalization during the infection of entomopathogenic fungus *M. robertsii*.

## Material and methods

### Fungal strains and culture conditions

*M. robertsii* strain ARSEF2575 was obtained from the Agricultural Research Service Collection of Entomopathogenic Fungi. The wild-type (WT) and transformants were routinely cultivated on potato dextrose agar (PDA, pH 5.6) (BD Difco, New Jersey, USA) at 25 °C. For growth analysis with different pH, the PDA or 1% casein medium was supplemented with the following buffers and adjusted to the indicated pH: 77 mM Na_2_HPO_4_, 60 mM citric acid (pH4.2); 30 mM Na_2_HPO_4_, 62 mM NaH_2_PO_4_, 80 mM NaCl (pH 6.8); 50 mM Tris-HCl, 125 mM NaCl (pH 9.4) [14]. For nutrient-rich cultivation, fungi were cultured on the complete medium (CM: 10 g/L glucose, 6 g/L NaNO_3_, 0.52 g/L KCl, 0.52 g/L MgSO_4_ · 7 H_2_O, 0.25 g/L KH_2_PO_4_, 1 g/L yeast extract, 2 g/L peptone, 1 g/L casamino acid, 1 ml/L trace elements, 1 ml/L vitamin supplement, pH 6). The minimal medium without glucose (MM: 6 g/L NaNO_3_, 0.52 g/L KCl, 0.52 g/L MgSO_4_ · 7 H_2_O, 0.25 g/L KH_2_PO_4_, 1 ml/L trace elements, 10 mg/L vitamin B1, pH 6 or 6.5) was used as a basal medium for nutrient studies [15]. The cuticle was dissected from *Tenebrio molitor* larvae and ground into powder, and fungal strains were cultivated on the MM supplied with 2% (w/v) cuticle powder for 2 to 10 days.

### Gene deletion and complementation

The *M. robertsii GDH2* gene (*MAA_02480*, *MrGDH2*) was deleted by homologous recombination via *Agrobacterium*-mediated fungal transformation as described previously [16]. In brief, the upstream and downstream flanking sequences were amplified from genomic DNA with primer sets UF/UR and DF/DR, respectively (Table S1). The PCR products were digested with the restriction enzymes *Bam* HI or *Bcu* I, and then inserted into the corresponding sites of the binary vector pDHt-bar (conferring resistance against ammonium glufosinate) for fungal transformation. For null mutant complementation, the 1350 bp of upstream sequence of *MrGDH2*, *MrGDH2* gene (3279 bp), and the 308 bp of downstream sequence were amplified with primers (Comp-F and Comp-R) containing *Xba* I. The PCR products were digested and inserted into the binary vector pDHt-ben (conferring resistance against benomyl) for fungal transformation with Δ*Mrgdh2* [17]. Total RNAs of mycelia collected from 3 days of cultivation in sabouraud dextrose broth (SDB, pH 5.6) were extracted for confirming the expression of *MrGDH2* gene by RT-PCR.

### Induction of appressorium and examination of cuticle penetration

The sterile cicada wings were immersed in conidial suspension containing 4–10 × 10^7^ conidia/mL for 30 s before being placed on the 0.8% agar plate as described previously [17]. Appressoria were examined and counted using a microscope after incubation for 18 h. To conduct the insect cuticle penetration assay, a segment of mealworm (*T. molitor*) abdominal cuticle was dissected [18]. A drop of 0.2 μL conidial suspension (1 × 10^7^ conidia/mL) was inoculated in the center of the sterilized cuticle. The cuticles were removed after 48 h of cultivation on the agar plate, and the penetrated mycelia were stained with lactophenol cotton blue to determine the diameter under a microscope (Nikon eclipse Ni-U, Tokyo, Japan).

### RNA extraction and quantitative real-time PCR (qRT-PCR) analysis

The WT strain was cultivated on PDA (pH 5.6) for 2 weeks, and conidia were collected for RNA extraction. Mycelia were harvested from 3 days of cultivation in SDB (pH 5.6). Appressoria were induced on the cicada wings, collected, and ground with liquid nitrogen for RNA extraction. Hyphal bodies were harvested from the hemolymph of 6^th^ instar *Spodoptera litura* larvae injected with conidia for 48 h. Total RNAs of conidia, mycelia, appressoria, and hyphal bodies were extracted, respectively, with TRIzol and reversely transcribed into cDNAs with HiScript III RT SuperMix reagent kit (Vazyme, Nanjing, China). Gene expressions were determined by qRT-PCR with specific primers (Table S1) and ChamQ Universal SYBR Color qPCR Master Mix (Vazyme, Nanjing, China) on an Applied QuantStudio 6 Flex Real-Time PCR System. Three biological replicates were performed, and quantification of transcript level of the target gene was conducted using the 2^−ΔΔCt^ method [19].

### Enzyme activity assays

The proteinase activity was detected by the azocasein assay [20]. In brief, azocasein was dissolved at a concentration of 5 mg/mL in a buffer containing 50 mM Tris (pH 7.5), 0.2 M NaCl, 5 mM CaCl_2_, 0.05% Brij 35, and 0.01% sodium azide. Appressoria were induced on the cicada wing or cellophane membrane for 36 h, collected, and ground with 10 mM PBS (phosphate buffered saline, pH 7.4) to extract total proteins. The supernatant (50 μL) was mixed with the azocasein solution (100 μL), and the mixture was incubated in a 37 °C water bath for 90 min. The reactions were stopped by trichloroacetic acid, and 50 μL of 1 M NaOH was added. The absorbance at 436 nm of released azo dye was determined with a spectrophotometer (FlexStation 3 microplate reader, Molecular Devices, California, USA). One unit of protease activity was defined as the amount of enzyme required to cause an increase in absorbance of 0.01 at 436 nm/min. Chitinase hydrolysis assay was performed on colloidal chitin (1%, wt/vol) medium containing 7 mM KCl, 2 mM MgSO_4_, 50 mM Na_2_HPO_4_-NaH_2_PO_4_ (pH 5.3), 0.05% (wt/vol) Triton X-100, and trace elements [14].

### Fungal growth in the hemolymph and fat body

The surface of *Galleria mellonella* larvae at the last instar was disinfected with 75% alcohol in a clean bench. Hemolymph was collected, and phenylthiourea (PTU) was added to inhibit melanization caused by exposure to air. Additionally, 1% kanamycin and 1% ampicillin were supplemented to prevent bacterial infections. Plasma (cell-free hemolymph) was harvested by centrifuged hemolymph at 2000 rpm for 10 min to remove hemocytes. Conidia were inoculated into hemolymph or plasma and grown for 5 to 7 days. The pH of the hemolymph or plasma after 6 days of fungal growth was indicated by adding phenol red. Fat bodies were dissected from sterilized *G. mellonella* larvae and washed in sterile water before placing in PBS containing 1% kanamycin and 1% ampicillin. Conidia were inoculated into PBS or PBS containing a layer of fat bodies and cultivated for 7 days.

### Insect bioassays

Insect bioassays were conducted using the last instar larvae of the mealworm beetle *T. molitor*. Conidia from PDA plates were used for topical infection by spraying 800 μL suspension of 10^7^ conidia/mL. For injection assays, 10 μL of an aqueous suspension containing 5 × 10^5^ conidia/mL were injected into the second proleg of each larva. Each treatment had three replicates with at least 15 larvae in each replicate, and the experiments were repeated three times. Larval mortality was recorded every 12 h, and the LT_50_ values were estimated by Kaplan–Meier analysis [21].

### Detection of total carbohydrate content

To determine the total sugars in the hemolymph of *G. mellonella* larvae, the anthrone method was performed as previously described [22]. Glucose was used for the standard curve. Briefly, hemolymph from different treated larvae groups was collected and centrifuged at 2000 rpm for 10 min, and plasma was collected. Anthrone solution was made by adding 0.1 g anthrone to 80% concentrated sulfuric acid. Aliquots (20 μL) of plasma samples were mixed with 1.98 mL water and 6 mL anthrone solution, and the mixtures were treated at 90 °C for 15 min. After cooling on ice, the absorbance of samples at 625 nm was measured in a spectrophotometer (FlexStation 3 microplate reader, Molecular Devices, California, USA).

### Statistical analysis

Graphical representation and analysis of the data were performed using GraphPad Prism 8.0 (GraphPad Software, Inc.). Multiple comparisons across strains were performed by one-way or two-way ANOVA with Tukey’s honest significance test (Tukey’s HSD) (***p < 0.001, **p < 0.01, *p < 0.05, and ns for *p* > 0.05). For multiple comparisons involving more than three samples, different letters indicate statistically significant differences (*p* < 0.05).

## Results

### MrGdh2 is responsible for alkalization during fungal growth on insect wing or cuticle powder

**Figure 1.**
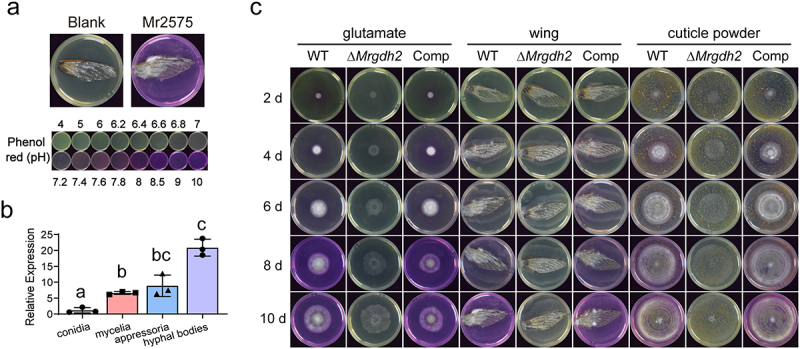
Fungal growth on medium containing insect wing or cuticle powder. (a) *M. robertsii* 2575 strain (WT) was able to alkalize ambient pH during growth on cicada wing. The wing treated with 0.05% tween solution was used as the blank control. 0.001% phenol red was supplied as pH indicator. The colorimetric response of 0.001% phenol red to pH was present. (b) The expression pattern of *MrGDH2* gene in conidia, mycelia, appressoria and hyphal bodies. Values are means ± SD of three replicates; different letters indicate significant differences (one-way ANOVA with Tukey’s test, *p* < 0.05). (c) Growth of the WT, Δ*Mrgdh2* and Comp strains on minimal medium containing 10 mM glutamate (left), cicada wing (middle) or 2% cuticle powder (right) for 2 to 10 days.

Consistent with a previous study demonstrating that *M. robertsii* was able to alkalinize the media [13], we also found that *M. robertsii* was able to alkalinize environmental pH when cultured on insect wing for 7 days (Figure 1(a)). Gdh2 has been proven to catalyze ammonia accumulation in *Colletotrichum* species during the alkalinization process [7]. We found that *MrGDH2* gene was expressed at the highest level in hyphal bodies (fungal hyphae in the hemocoel), followed by in appressoria and mycelia (Figure 1(b)). To evaluate the function of Gdh2 in *M. robertsii*, a deletion mutant (Δ*Mrgdh2*) and a complement strain (Comp) were created (Figure S1). We found that the Δ*Mrgdh2* mutant failed to catalyze glutamate deamination, leading to its incapacity to generate ammonia and alkalization in media containing only insect wings or cuticle powders as well as in the minimal medium (MM) with 10 mM glutamate (Figure 1(c)).

### The full virulence of *M.* *robertsii* requires the activity of MrGdh2

**Figure 2.**
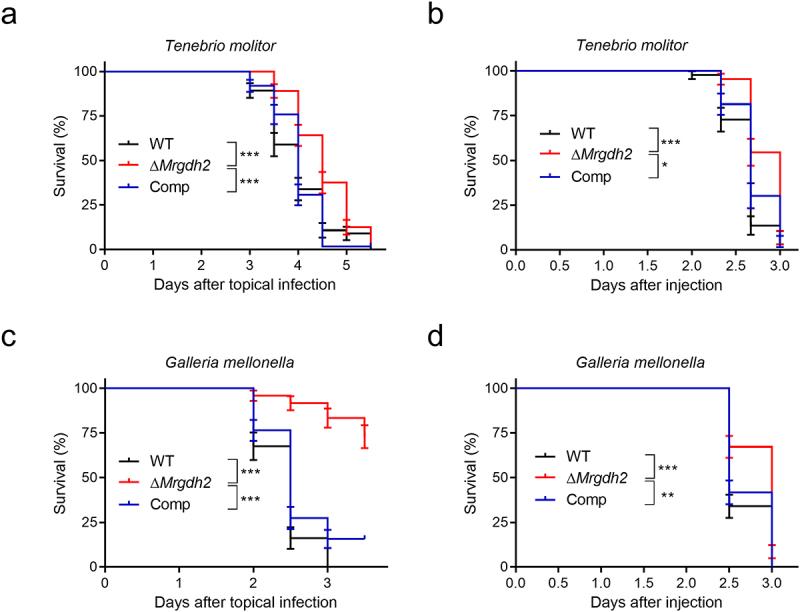
Insect bioassays. (a, c) survival of *T. molitor* (a) and *G. mellonella* (c) larvae following topical infection with spore suspensions. Control insects were treated with 0.05% tween 20. (b, d) survival of *T. molitor* (b) and *G. mellonella* (d) larvae following injection with conidia. Control insects were injected with 0.05% tween 20. Asterisk indicates significant differences (Kaplan−Meier analysis, ***p < 0.001, **p < 0.01, and *p < 0.05).

To investigate the effect of MrGdh2 activity on pathogenicity, bioassays were performed by topically applying conidia to insect cuticle (cuticle-passing infection) or injecting conidia into hemocoel (cuticle-bypassing infection). The survival patterns of inoculated larvae showed that the lethal action of Δ*Mrgdh2* was delayed during cuticle-passing or cuticle-bypassing infections (Figure 2). As a topical application against *T. molitor*, Δ*Mrgdh2* had a substantially longer LT_50_ (4.52 ± 0.076 days) than the WT (4.0 ± 0.091 days, *p* < 0.001) or Comp (4.0 ± 0.061 days, *p* < 0.001) (Figure 2(a)). Topical infection against *G. mellonella* larvae also revealed a reduced virulence in the Δ*Mrgdh2* mutant when compared to the WT and Comp (Figure 2(c)). When *T. molitor* larvae were infected via direct injection, the Δ*Mrgdh2* mutant also had a longer LT_50_ (2.83 ± 0.036 days) than the WT (2.61 ± 0.034 days, *p* < 0.001) (Figure 2(b)), and similar results were observed when *G. mellonella*, *Spodoptera litura*, and *Bombyx mori* larvae were infected (Figures 2(d) and S1(c-d)). These results suggest that MrGdh2 activity is essential for the full virulence of *M. robertsii* against insect hosts.

### Deletion of MrGDH2 has no effect on appressorium formation and host cuticle penetration ex vivo

**Figure 3.**
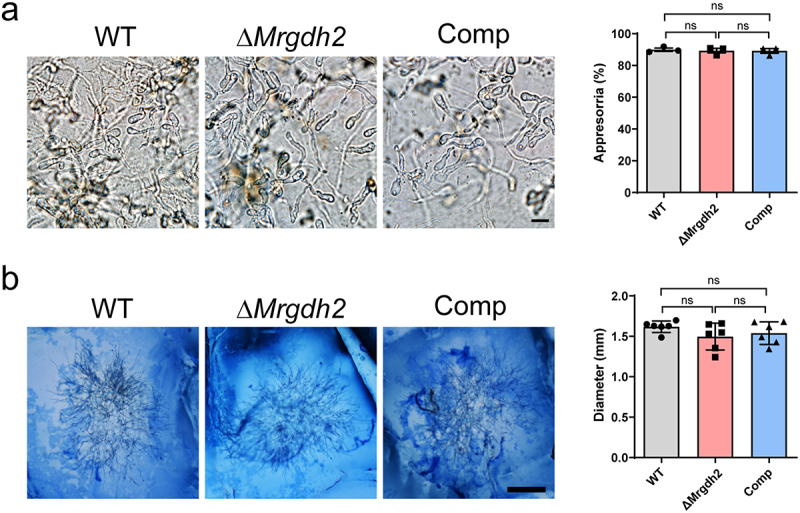
Appressorium formation and penetration analyses. (a) Comparison of the morphology and formation rate of appressoria induced on the cicada wings for 18 h. Values are means ± SEM of replicates. Bar, 5 μm. (b) Fungal penetration on the cuticle of *T. molitor* larvae. The diameter of the penetrated mycelia was measured after inoculating conidia on the cuticle for 48 h. Values represent means ± SD of replicates. Bar, 500 μm. No significant difference (ns) was observed between groups based on one-way ANOVA with Tukey’s test.

Deletion of *GHD2* decreased the formation of appressoria and resulted in reduced virulence in the plant pathogens [7,8,23]. To explore the reason for the reduced pathogenicity of Δ*Mrgdh2*, we examined its capacity to generate appressorium. Interestingly, the morphology and formation ratio of appressoria on the cicada wings remained unchanged among the WT, Δ*Mrgdh2* and Comp strains (Figure 3(a)).

Cuticle-degrading enzyme is a pathogenic factor for fungi to breakdown cuticle, and the expression and activity of enzymes are affected by changes in pH [12,24]. Compared to the WT and complement strains, the Δ*Mrgdh2* mutant showed no significant difference in the protease activity of appressoria induced on the cicada wing and cellophane membrane (Figure S2a-b). Compared to the Δ*MrPacC* mutant which was disrupted in chitinase activity [14], the WT, Δ*Mrgdh2* and Comp strains showed no difference in chitinase activity based on the growth in 1% colloidal chitin medium (Figure S2c).

To further explore the role of MrGdh2 in fungal penetration, we examined its capacity to penetrate the insect cuticle and wing. The Δ*Mrgdh2* mutant displayed the same capacity as the WT and Comp strains to penetrate the larval cuticle of *T. molitor* and cicada wing (Figures 3(b) and S3). These findings suggest that Ggh2-dependent alkalization is not required for *M. robertsii* to produce appressoria or penetrate insect cuticle ex vivo.

### MrGdh2 is vital for amino acid-dependent fungal growth and reproduction during carbon deprivation

**Figure 4.**
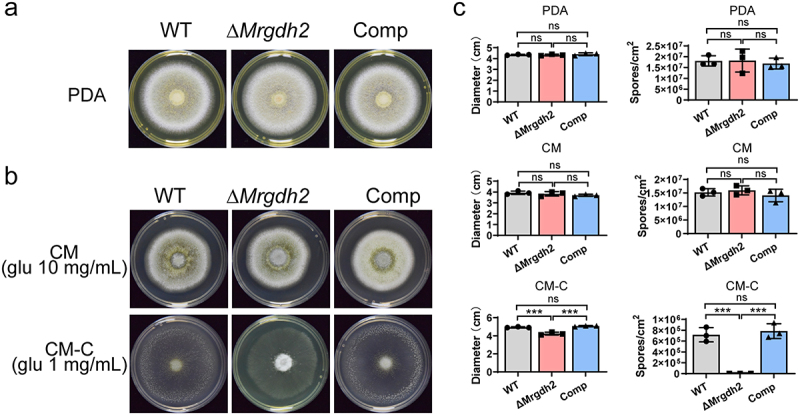
Growth and conidiation of *M. robertsii* strains on media with different nutrition. (a) Growth of the WT, Δ*Mrgdh2* and Comp strains for 10 days on PDA plates. (b) Fungal strains grown for 10 days on complete medium containing 10 mg/mL glucose (CM) or 1 mg/mL glucose (CM-C). 0.001% phenol red was supplied as pH indicator. (c) The diameter and spore production of the colonies were analyzed. Values represent means ± SD of three replicates, the comparisons were performed by on one-way ANOVA with Tukey’s test (***p < 0.001, **p < 0.01, *p < 0.05 and ns for *p* > 0.05).

The WT, Δ*Mrgdh2,* and Comp strains exhibited identical colony diameter and conidial production on rich media such as PDA and complete medium (CM) (Figure 4(a-b)). Furthermore, these strains displayed no difference in conidial yield and pH alteration when grown on PDA buffered to pH 4.2, 6.8, and 9.4 (Figure S4). However, a reduction in glucose content from 10 mg/mL to 1 mg/mL impaired the ability of the Δ*Mrgdh2* mutant to alkalize the medium. The media inoculated with the WT and Comp strains exhibited a pH around 7.4; however, following cultivation with the Δ*Mrgdh2* mutant, the medium pH only reached approximately 6.4 (Figure 4(b)). The colony of Δ*Mrgdh2* was just slightly smaller than the WT and Comp strains, but a significantly lower number of spores emerged from the Δ*Mrgdh2* mutant (Figures 4(b-c)).

**Figure 5.**
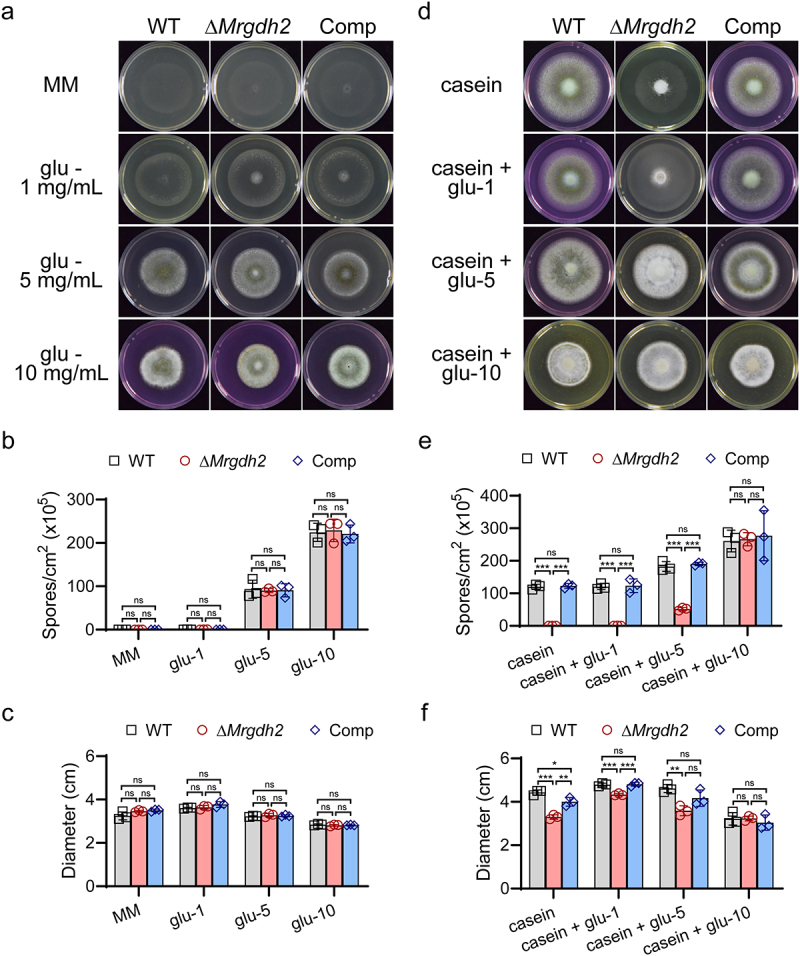
*M. robertsii* Gdh2 is required for amino acid-dependent alkalization during glucose deprivation. Minimal medium without glucose (MM) was used as a basal medium. The WT, Δ*Mrgdh2* and Comp strains were cultivated for 10 days on MM or hydrolyzed casein media supplemented with different concentrations of glucose (a, d). 0.001% phenol red was supplied as pH indicator. Conidial production (b, e) and diameter (c, f) of colonies were analyzed. Values are means ± SD of three replicates, the differences across strains within each medium were analyzed by one-way ANOVA with Tukey’s test (***p < 0.001, **p < 0.01, *p < 0.05 and ns for *p* > 0.05).

To further explore the effect of nutrients on MrGdh2 activity, a minimal medium without glucose (MM) was used as a basal medium. Only sparse vegetative mycelia of the WT, Δ*Mrgdh2,* and Comp strains were maintained on the MM medium; however, the amounts of mycelia and conidia were increased with increasing concentrations of glucose in the medium (Figure 5(a-b)). There was no difference in colony morphology or conidial yield among the WT, Δ*Mrgdh2* and Comp strains during growth under these conditions (Figure 5(a-c)). Interestingly, Δ*Mrgdh2* produced fewer aerial mycelia and conidia, and failed to alkalize MM with 1% hydrolyzed casein as an amino acid resource (Figures 5(d-f) and S5). The Δ*Mrgdh2* strain also failed to alter the pH in casein medium with 1 or 5 mg/mL glucose (Figure 5(d)). Although aerial mycelia and conidia of the Δ*Mrgdh2* strain were more abundant when grown in media with 5 mg/mL glucose than 1 mg/mL (Figure 5(a,d)), conidial yield of the mutant strain was decreased in casein media with both 1 and 5 mg/mL glucose when compared to the WT and Comp strains (Figure 5(e,f)). No differences were observed in fungal growth and conidia yield among the three strains when grown in casein medium with 10 mg/mL glucose (Figure 5(d-f)), and none of these strains altered the pH of the medium (Figure 5(d)), suggesting that *M. robertsii* does not necessitate deamination of amino acids to produce energy when glucose is sufficient.

**Figure 6.**
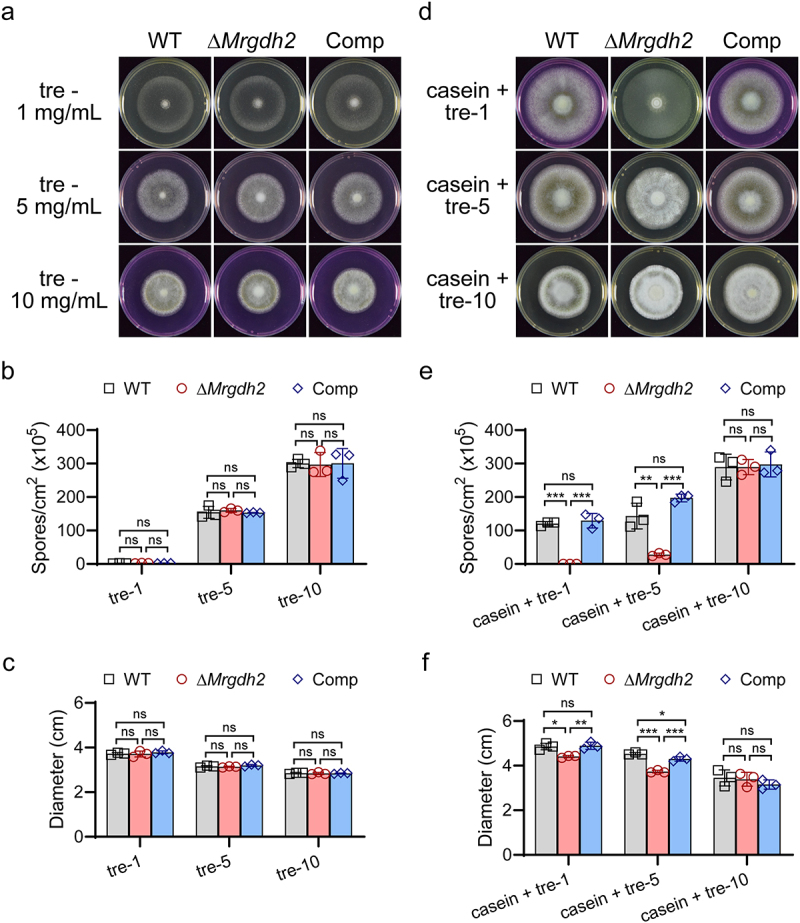
*M. robertsii* Gdh2 is required for amino acid-dependent alkalization during trehalose deprivation. The WT, Δ*Mrgdh2* and Comp strains were cultivated for 10 days on MM or hydrolyzed casein media supplemented with different concentrations of trehalose (a, d). 0.001% phenol red was supplied as pH indicator. Conidial production (b, e) and diameter (c, f) of colonies were analyzed. Values are means ± SD of three replicates, the differences across strains within each medium were analyzed by one-way ANOVA with Tukey’s test (***p < 0.001, **p < 0.01, *p < 0.05 and ns for *p* > 0.05).

Trehalose is a major circulatory sugar in insect hemolymph [25]. Similar outcomes to those observed with glucose were achieved when trehalose served as the carbon source (Figure 6). When the WT, Δ*Mrgdh2* and Comp strains were supplied with 1% gelatin as a nitrogen source [26], the colony diameter and conidial yield of Δ*Mrgdh2* strain were also decreased in gelatin media containing 0, 1, or 5 mg/mL glucose or trehalose (Figure S6). In addition, the growth defects of Δ*Mrgdh2* in casein media were independent of the environmental pH (Figure S7). These results suggest that during carbon deprivation, fungal development requires the MrGdh2 activity to convert amino acids to produce α-ketoglutarate and NADH, both are vital intermediates for energy metabolism [27].

### The activity of MrGdh2 is required for efficient fungal colonization in insect hemolymph

**Figure 7.**
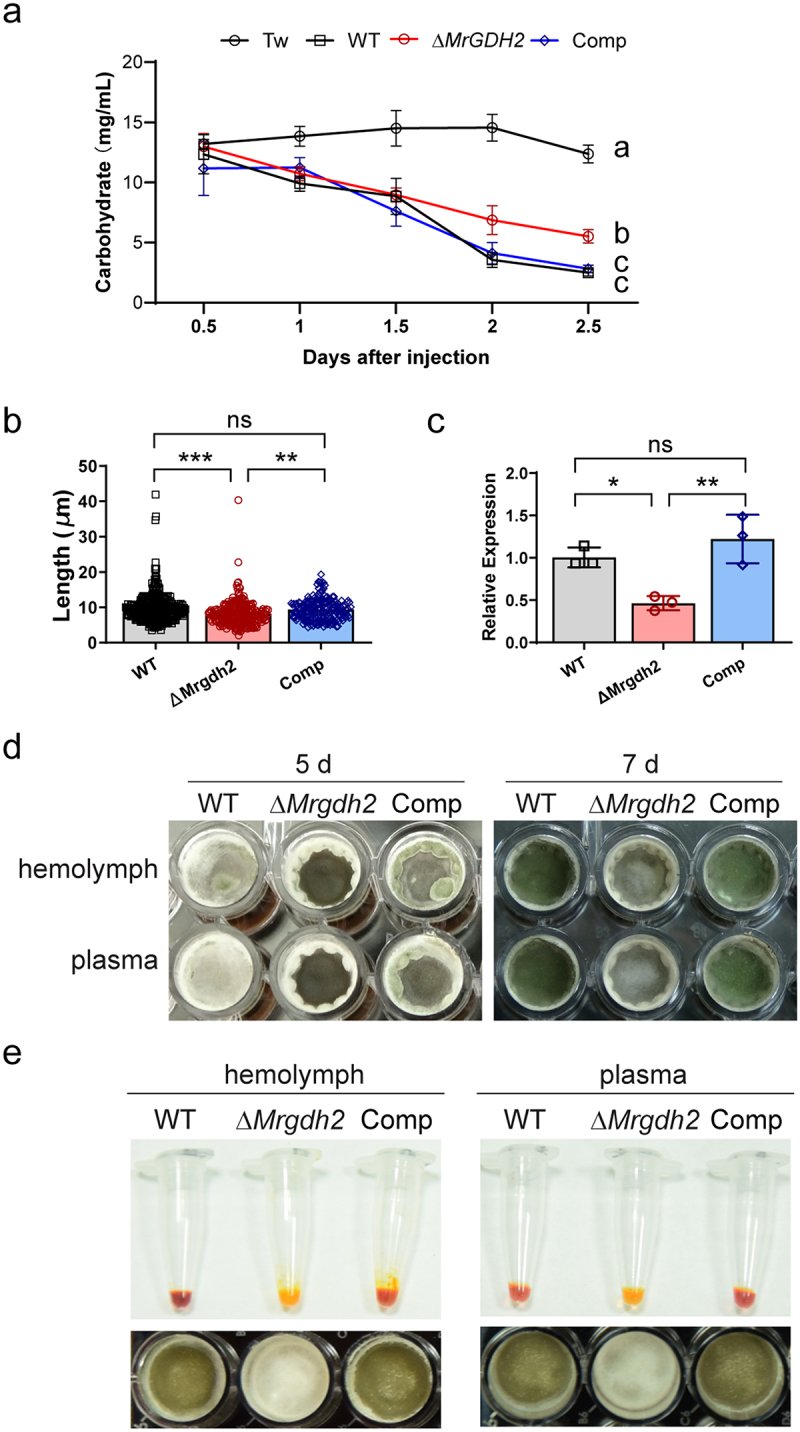
Hemolymph carbohydrate contents during *M. robertsii* infection and fungal growth in the hemolymph. (a) Carbohydrate contents in the hemolymph of *G. mellonella* larvae infected by the WT, Δ*Mrgdh2* or Comp strain were quantified. Control larvae were treated with 0.05% tween 20 (tw, control). Values are means ± SD of three replicates. The comparisons across infections by different strains were performed by two-way ANOVA with Tukey’s test (also see Table S2), with different letters indicating statistically significant differences (*p* < 0.05). (b) The length of hyphal bodies in the hemolymph. Hyphal bodies emerged in the hemolymph of *S. litura* larvae were observed by light microscope after injection of the WT, Δ*Mrgdh2* or Comp strain for 2 days. (c) The expression level of *MrPacc* in hyphal bodies of the WT, Δ*Mrgdh2* or Comp strain was analyzed by qRT-PCR with *EF-1* as a reference gene. Values are means ± SD, the comparisons were performed by one-way ANOVA with Tukey’s test (***p < 0.001, **p < 0.01, *p < 0.05 and ns for *p* > 0.05). (d) The growth of the WT, Δ*Mrgdh2* and Comp strains in the hemolymph or plasma for 5 and 7 days. (e) The pH of the hemolymph or plasma was indicated by adding phenol red after inoculating the WT, Δ*Mrgdh2* or Comp strain for 6 days.

Both immune defense and pathogen replication within the host involve substantial energy demands [28]. We proposed that *M. robertsii* infection may cause carbon deprivation in insect hemolymph. We found that total carbohydrate content in the hemolymph of uninfected *G. mellonella* larvae was around 13 mg/mL (Figure 7(a)). However, the carbohydrate content in the hemolymph of larvae infected with *M. robertsii* was significantly reduced, dropping to 10 mg/mL at 1 d (1 day), 8.8 mg/mL at 1.5 d, 3.5 mg/mL at 2 d, and 2.5 mg/mL at 2.5 d after injection of *M. robertsii* conidia (Figure 7(a)). The carbohydrate contents in the hemolymph of Δ*Mrgdh2*-infected larvae were also decreased but were slightly higher at 2 d and 2.5 d after infection compared to the WT and Comp strains (Figure 7(a), Table S2).

After penetration into insects, fungal hyphae secrete enzymes and metabolites to inhibit host immunity or take in host nutrients, which facilitate the successful colonization of fungi in the hemocoel [29]. The number of hyphal bodies was similar between the WT and Δ*Mrgdh2* mutant in the hemolymph of *S. litura* larvae (Figure S8a); however, the length of hyphal bodies of the Δ*Mrgdh2* mutant was shorter than the WT and Comp strains in the hemolymph (Figures 7(b), S8(b)), indicating that MrGdh2 participated in fungal growth in insect hemocoel. The pH-responsive PacC transcription factor contributes to virulence by regulating blastosphere budding in *M. rileyi* [30]. We found that the expression of MrPacC in hyphal bodies of the Δ*Mrgdh2* mutant was lower compared to the WT and Comp strains (Figure 7(c)).

To further investigate the impact of MrGdh2 activity during fungal colonization in host hemocoel, hemolymph was used as nutrition source for fungal growth. The hemolymph or plasma (cell-free hemolymph) was entirely covered by the white mycelia of the WT and Comp strains after 5 days of cultivation, whereas the growth of Δ*Mrgdh2* was delayed (Figure 7(d)). The conidia yield of Δ*Mrgdh2* was also notably reduced after cultivating for 7 days (Figure 7(d)). Correspondingly, Δ*Mrgdh2* failed to alkalize hemolymph or plasma (Figure 7(e)). In addition, there was no notable difference in hyphal growth between the WT and Δ*Mrgdh2* after 7 days of cultivation in PBS supplemented with fat bodies from *G. mellonella* larvae (Figure S8c). These results imply that MrGdh2 is crucial for fungal colonization in insect hemocoel.

## Discussion

Fungal infections are often associated with pH changes in the surrounding host tissues [31]. Plant fungal pathogens induce alkalinization through regulated release of ammonia, and in certain phytopathogens by secreting small regulatory peptides that mimic plant rapid alkalinizing factors (RALFs) [3,4]. *M. robertsii* can alkalinize artificial medium by production of ammonia [13]. Given that yeast Gdh2 mediates glutamate deamination to α-ketoglutarate and ammonia [5], MrGdh2 also participated in deamination of glutamate (Figure 1(c)). A previous study reported that *M. robertsii* was able to alkalinize insect cuticle during infection [12]. We showed that *M. robertsii* alkalinized the medium containing wing or cuticle powder as a nutrition source after growth for 7 days, but this ability was impaired upon *MrGDH2* deletion (Figure 1(c)).

In plant pathogens *M. oryzae* and *Colletotrichum*, *GDH2* deletion disrupts appressorium formation and virulence [7,8,23]. Although MrGdh2 activity is required for virulence during cuticle-passing bioassay (Figure 2(a)), the abilities to form appressorium and penetrate cuticle were not affected in Δ*Mrgdh2* (Figures 3, S3). Ambient pH is an important determinant for cuticle-degrading enzymes to penetrate insect cuticle by *M. robertsii* [11,12]. Deleting *MrGDH2* resulted in no changes in protease and chitinase activities (Figures 3(b), S2, S3). The pH around the wing remained unchanged during the first 48 h post-inoculation (hpi), a period coinciding with appressorium formation (Figure 1(c)), suggesting that MrGdh2 appears to be dispensable for appressorium formation and cuticle penetration during early infection but essential for subsequent fungal growth under nutrient-limited conditions. *Metarhizium* was likely originated from plant endophytes or pathogens [32]. However, Gdh2 contributes differently to appressorium formation between *Metarhizium* and plant pathogens, suggesting distinct mechanistic adaptations in host surface recognition and infection strategies.

Carbon limitation triggers extracellular accumulation of ammonia and alkalinization in several plant and human fungal pathogens [9,23]. For example, *Colletotrichum gloeosporioides* alkalinizes tryptone-containing media under carbon deprivation [23]. Unlike the WT, Δ*Mrgdh2* failed to alkalize hydrolyzed casein media supplemented with low glucose/trehalose (0–5 mg/mL; Figures 5–6) or CM with 1 mg/mL glucose (Figure 4(b)). However, neither strain alkalinized PDA, CM, MM, or 1% casein medium with 1% carbon source (Figures 4–6), suggesting that MrGdh2 mediates glutamate deamination specifically during carbon shortage when amino acids are available, with alkalization occurring as a metabolic byproduct of amino acid utilization. *C. albicans* Gdh2 expression is repressed by glucose [9]. We also found that alkalization of the casein media disappeared after increasing glucose content to 10 mg/mL (Figure 5). Intriguingly, both the WT and Δ*Mrgdh2* strains alkalized amino acid-free MM containing 10 mg/mL glucose/trehalose (Figure 5(a), 6(a)), but this ability was lost upon casein addition (Figure 5(d), 6(d)). These findings suggest that glucose metabolites may undergo enhanced catalysis to compensate for the nitrogen deficiency in the absence of amino acids, resulting in alkalinization.

In *C. albicans*, Gdh2 is required for growth using amino acids as sole carbon and nitrogen sources [9]. Similarly, in *M. robertsii*, MrGdh2 is vital for mycelia growth (particularly aerial mycelia) under such conditions. Moreover, *MrGDH2* deletion significantly impaired conidiation (Figure 5(d), 6(d)). Aerial hyphae growth depends on vegetative mycelia for nutrients, and serves as the foundation of conidiation [32,33]. Accordingly, increasing the carbon source enhanced both aerial mycelia growth and conidia yield in *M. robertsii* (Figure 5,6). Gdh2 catalyzes the deamination of glutamate to α-ketoglutarate, a key metabolic intermediate required for many biological processes and linking amino acid metabolism to the TCA cycle [5,6]. In Δ*Mrgdh2*, loss of MrGdh2 function abolished α-ketoglutarate production from amino acids, and carbon limitation halts the TCA cycle, resulting in defects in both aerial mycelia growth and conidiation.

*C. albicans gdh2*^−/−^ mutants were as virulent as the wild-type strain [9]. However, loss of *MrGDH2* led to reduced virulence upon hemocoel injection (Figure 2(b-c)), indicating that MrGdh2 is involved in host colonization within insect hemocoel. Both immune defense and pathogen replication require substantial energy during infection [28], and hemolymph-derived nutrients vary across host-parasite systems [34,35]. For instance, parasitism of *Heliothis virescens* larvae by *Microplitis croceipes* but not *Cardiochiles nigriceps* resulted in an increase in host hemolymph trehalose concentration [36], whereas parasitization of *Pimpla turionellae* in *G. mellonella* pupae reduced the pupal hemolymph carbohydrate at 24 h postoviposition [37]. During *M. robertsii* infection, *G. mellonella* hemolymph carbohydrates dropped from 13 to 2.5 mg/mL (Figure 7(a)), suggesting carbon limitation. Amino acids are particularly important nutrients that are central to the host and bacterial pathogen interaction [37]. *MrGDH2* expression peaks in hyphal bodies (Figure 1(b)); its deletion shortened hyphal bodies and delayed growth in hemolymph/plasma (Figures 7(b), S8(c)). As hemolymph is rich in amino acids [38,39], we propose that amino acids serve as critical nutrients upon carbon source depletion during *M. robertsii* infection, and MrGdh2 facilitates host colonization via amino acid catabolism.

The pH-responsive PacC transcription factor is required for environmental fitness, development, and virulence of different fungal pathogens [30,40]. In *Metarhizium*, PacC contributes to virulence by regulating cuticle penetration and immune evasion in *M. acridum* and *M. robertsii*, as well as by regulating blastosphere budding in *M. rileyi* [14,30,41]. *MrPacC* expression in appressoria of Δ*Mrgdh2* was similar to that in the WT and Comp strains (Figure S9), while it was downregulated in hyphal bodies of Δ*Mrgdh2* (Figure 7(c)), implying a potential link between Gdh2 and PacC in blastosphere growth. Further studies are needed to elucidate this interaction.

Our study revealed that Gdh2 activity is required for virulence by facilitating fungal colonization in insect hemolymph in the entomopathogenic fungus *M. robertsii*, which is distinct from the established models of plant and human fungal infections. These findings advance our understanding of the diverse mechanisms of fungal infection across various hosts.

## Supplementary Material

Table S2.xlsx

Supplementary Material revise2.docx

Figure S6.tif

Figure S7.tif

Figure S4.tif

## Data Availability

All supporting data are openly available in figshare: https://doi.org/10.6084/m9.figshare.29209601.

## Abbreviations

Gdh2: glutamate dehydrogenase 2
TCA: tricarboxylic acid
Comp: complement
MM: minimal medium
CM: complete medium
RALFs: small regulatory peptides that mimic plant rapid alkalinizing factors.

## Supporting information

(Figure S1) Verification of transformants and larvae survival assays. (Figure S2) Assays for protease activity and chitinase activity. (Figure S3) Wing penetration assays. (Figure S4) Growth of *M. robertsii* strains on PDA buffered to various pH. (Figure S5) The cross section of fungal colonies. (Figure S6) *M. robertsii* strains cultivated on gelatin media with various carbon contents. (Figure S7) Growth of *M. robertsii* strains on casein media buffered to various pH. (Figure S8) Phenotypes of fungal strains grown in hemolymph or medium supplemented with insect fat bodies. (Figure S9) Relative expressions of *MrPacC* in appressoria. (Table S1) Primers used in this study. (Table S2) Two-way ANOVA results of Figure 7a.
