# Supplementary material for: Glutamate dehydrogenase 2 is required for virulence by facilitating fungal growth in the host hemocoel
Source: Virulence. 2025 Nov 26;16(1):2591402. doi: 10.1080/21505594.2025.2591402 (PMC12667647; doi:10.1080/21505594.2025.2591402)
Supplement: Supplementary Material revise2.docx [file KVIR_A_2591402_SM4622.docx]

**Supplementary data**

**Glutamate dehydrogenase 2 is required for virulence**

**by facilitating fungal growth in the host hemocoel**

Yuzhen Lu ^a^, Denghui Wu ^a^, Jiawei Hu ^a^, Guojun Peng ^a^, Jielai Zhong ^a^, Yan Liu ^b^, Jing Li ^a^,

Qiang Gao ^b*^, and Xiao-Qiang Yu ^a*^

1. Guangdong Provincial Key Laboratory of Insect Developmental Biology and Applied Technology, Guangzhou Key Laboratory of Insect Development Regulation and Application Research, Institute of Insect Science and Technology, School of Life Sciences, South China Normal University, Guangzhou 510631, China.
2. School of Biomedical Sciences, Hunan University, Changsha, Hunan, 410082, China

# Corresponding authors.

Email addresses: [xqyu@m.scnu.edu.cn](mailto:xqyu@m.scnu.edu.cn), [qianggao@hnu.edu.cn](mailto:qianggao@hnu.edu.cn)

**Supplementary data**

**Figure S1.** Verification of transformants and larvae survival assays.

**Figure S2.** Assays for protease activity and chitinase activity.

**Figure S3.** Wing penetration assays.

**Figure S4.** Growth of *M. robertsii* strains on PDA buffered to various pH.

**Figure S5.** The cross section of fungal colonies.

**Figure S6.** *M. robertsii* strains cultivated on gelatin media with various carbon contents.

**Figure S7.** Growth of *M. robertsii* strains on casein media buffered to various pH.

**Figure S8.** Phenotypes of fungal strains grown in hemolymph or medium supplemented with insect fat bodies.

**Figure S9.** Relative expressions of *MrPacC* in appressoria.

**Table S1.** Primers used in this study.

**Table S2.** Two-way ANOVA results of Figure 7a.


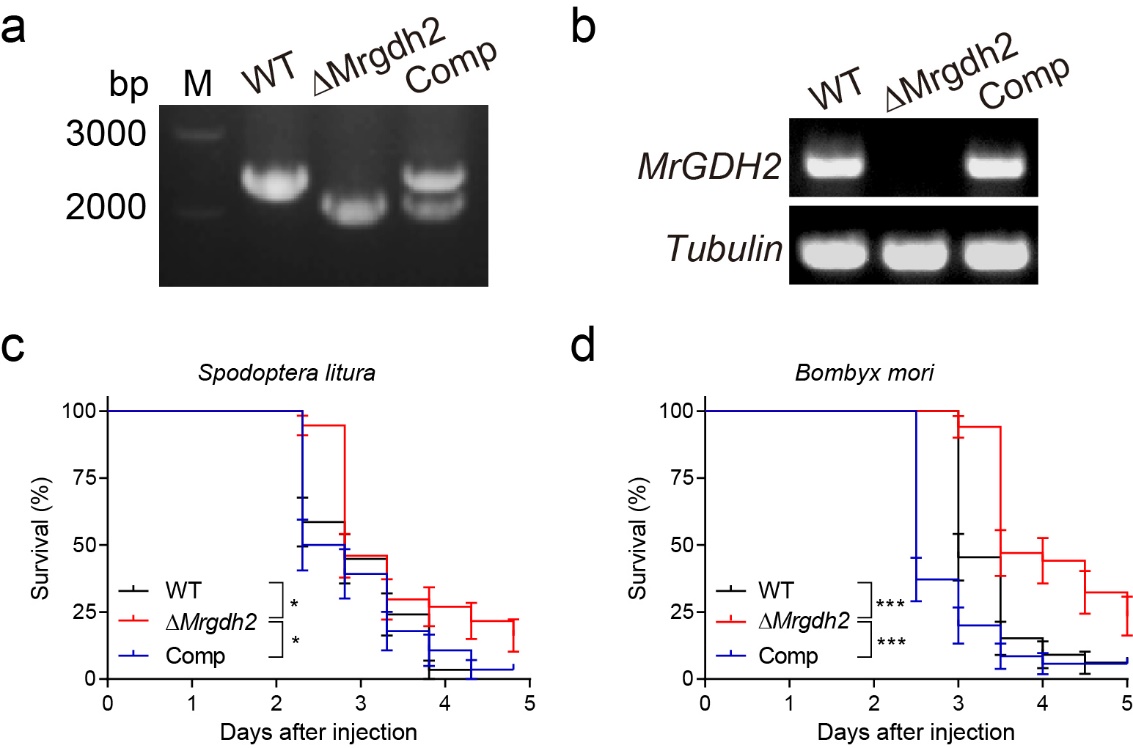


**Figure S1.** Verification of transformants and larvae survival assays. (a) Confirmation of Δ*Mrgdh2* and complement (Comp) strains by PCR using gDNA and GDH2-Test primers. (b) Verification of Δ*Mrgdh2* and Comp strains by RT-PCR analysis of *MrGDH2* expression in mycelia cultured for 3 days in SDB. (c-d) Survival of *Spodoptera litura* (c) and *Bombyx mori* (d) larvae following injection with spores. Asterisk indicates significant differences (Kaplan-Meier analysis, ****p* < 0.001, and **p* < 0.05).

**
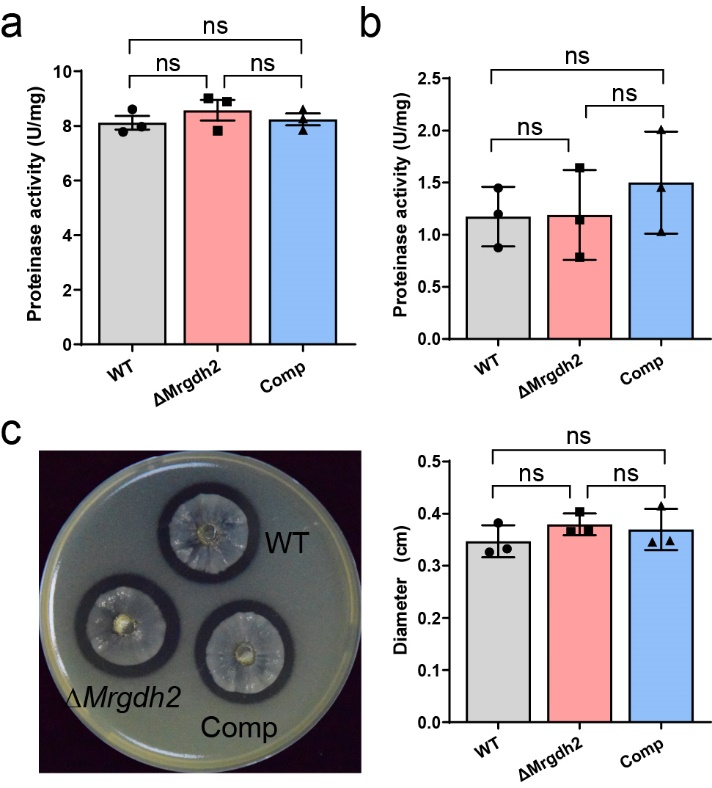
**

**Figure S2.** Assays for protease activity and chitinase activity. (a-b) Total protease activity of appressoria induced on cicada wing (a) or cellophane membrane (b) for 36 h was monitored by azocasein hydrolysis. (c) Chitinase activity was detected on a basal salt medium agar supplemented with 1% colloidal chitin as the sole carbon and nitrogen source for 12 days (left) and the size of the hydrolysis zone was measured (right). Values represent means ± SD of replicates. No significant difference (ns, *p* > 0.05) was observed between groups based on one-way ANOVA with Turkey’s test.


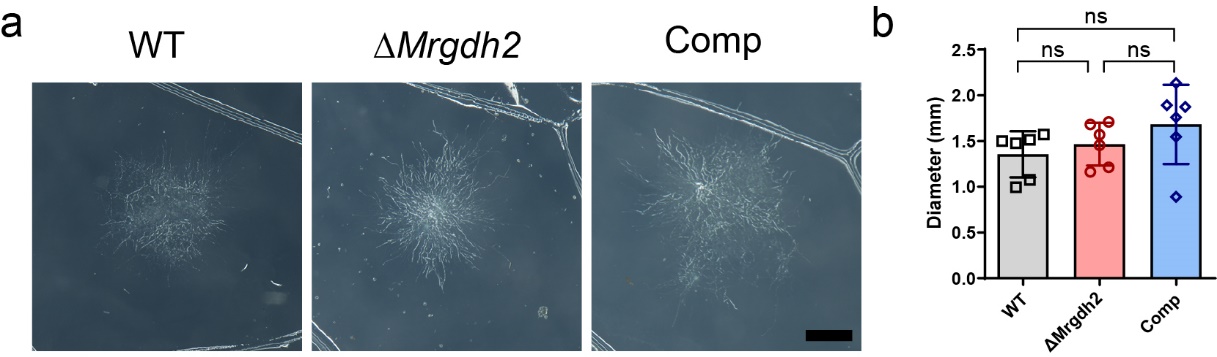


**Figure S3.** Wing penetration assays. (a) The cicada hind wings lined on the MM medium were inoculated with fungal strains in the middle for 50 h and then removed. Bar, 500 μm. (b) The diameter of the penetrated mycelia was measured after removing the wings. Values are means ± SD. No significant difference (ns, *p* > 0.05) was observed between groups based on one-way ANOVA with Turkey’s test.


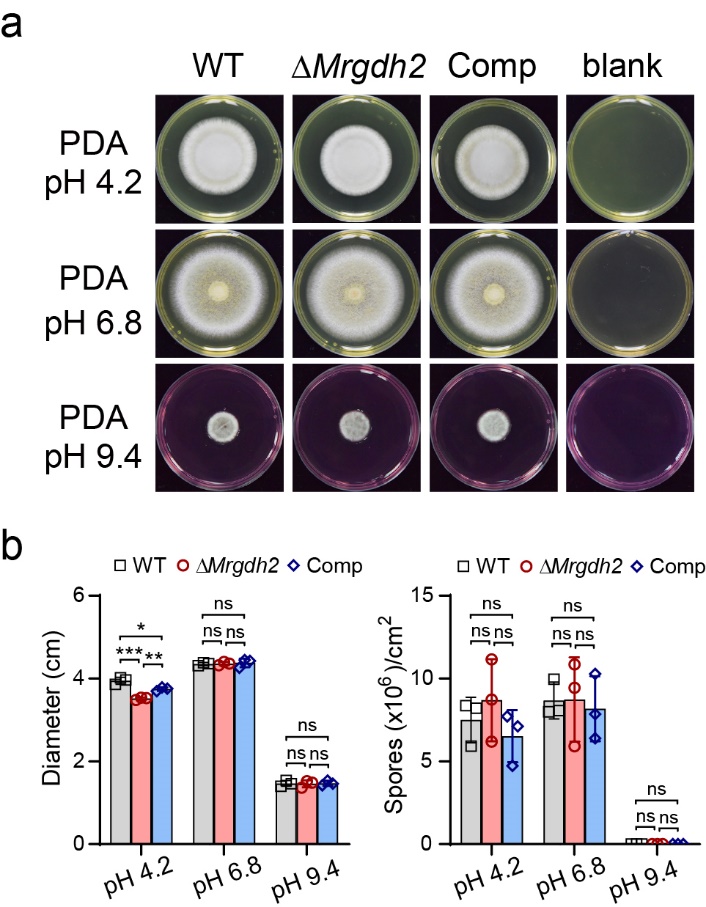


**Figure S4.** Growth of *M. robertsii* strains on PDA buffered to various pH. (a) The WT, Δ*Mrgdh2* and Comp strains were cultivated for 10 days on PDA buffered to pH 4.2, 6.8 and 9.4, respectively. (b) The diameter and spore production of the colonies were analyzed. Values are means ± SD of three replicates, the differences across strains within each medium were analyzed by one-way ANOVA with Tukey’s test (****p* < 0.001, ***p* < 0.01, **p* < 0.05, and ns for *p* > 0.05).

**
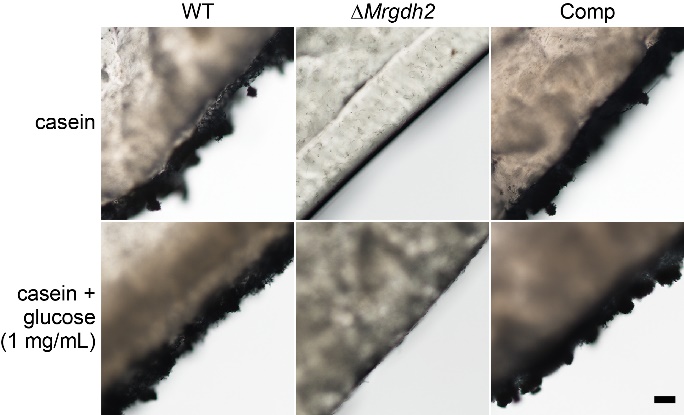
**

**Figure S5.** The cross section of fungal colonies. The WT, Δ*Mrgdh2* and Comp strains were cultivated for 10 days on hydrolyzed casein media without or supplemented with 1 mg/mL glucose. Aerial mycelia and spore clusters were obvious at the edge of the WT and Comp colonies, but were not observed in Δ*Mrgdh2* colonies. Bar, 100 μm.

**
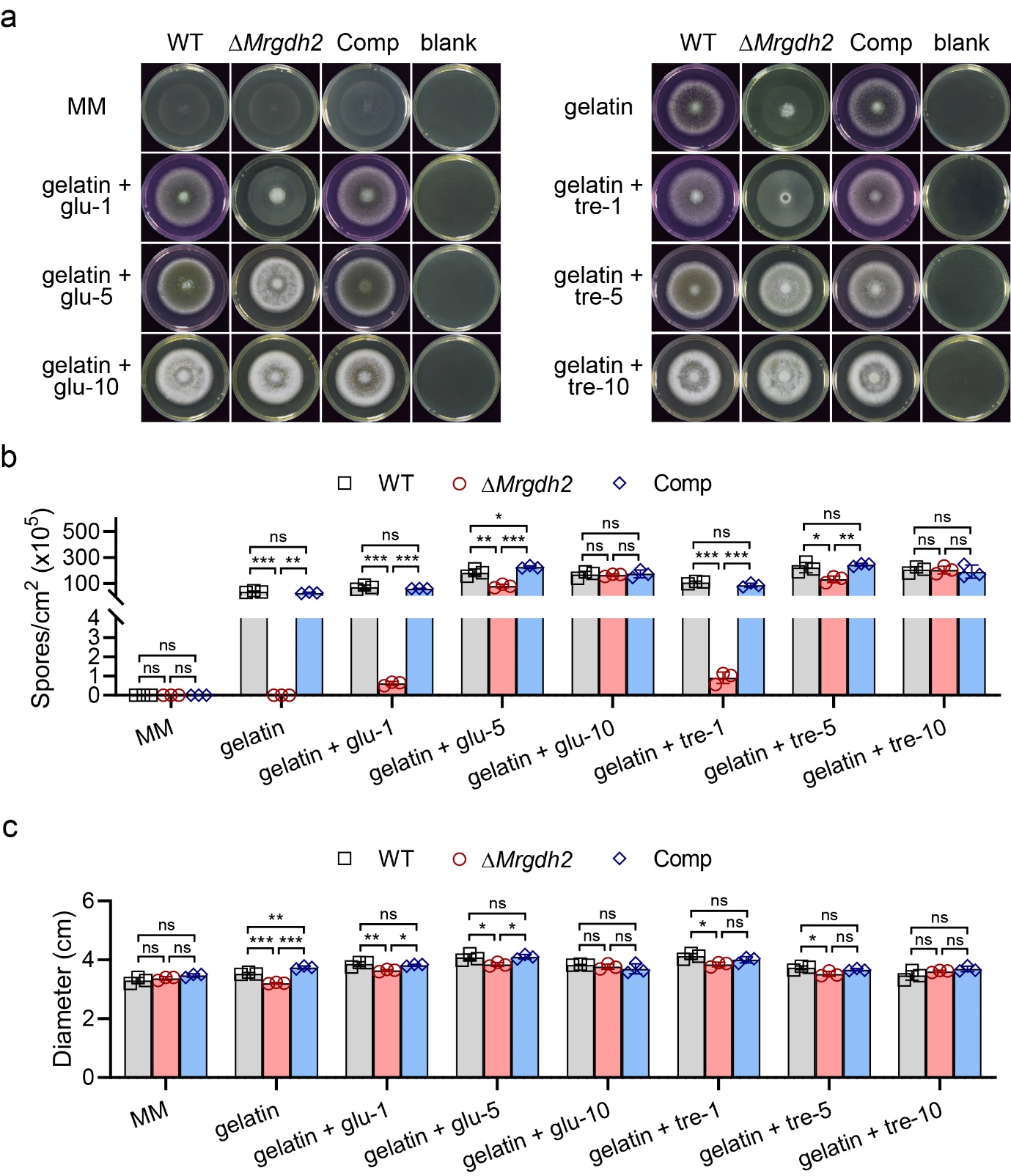
**

**Figure S6.** *M. robertsii* strains cultivated on gelatin media with various carbon contents. The WT, Δ*Mrgdh2* and Comp strains were cultivated for 10 days on MM or gelatin media supplemented with different concentrations of glucose or trehalose. 0.001% phenol red was supplied as pH indicator. Values are means ± SD of three replicates, the differences across strains within each medium were analyzed by one-way ANOVA with Tukey’s test (****p* < 0.001, ***p* < 0.01, **p* < 0.05, and ns for *p* > 0.05).


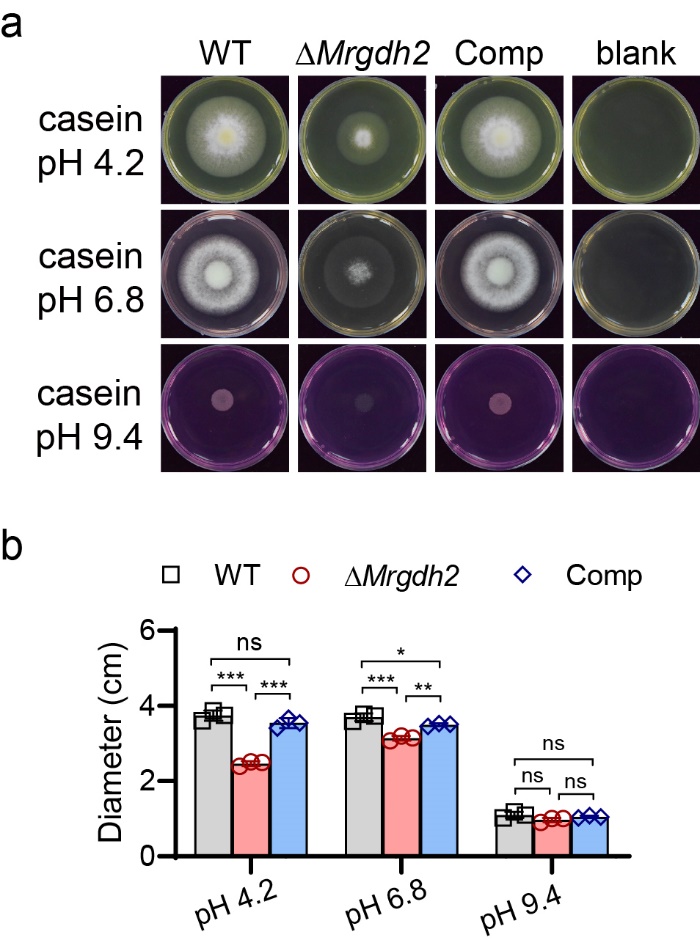


**Figure S7.** Growth of *M. robertsii* strains on casein media buffered to various pH. (a) The WT, Δ*Mrgdh2* and Comp strains were cultivated for 10 days on 1% casein medium buffered to pH 4.2, 6.8 and 9.4, respectively. No spore was produced in the media. (b) The diameter of the colonies was analyzed. Values are means ± SD of three replicates, the differences across strains within each medium were analyzed by one-way ANOVA with Tukey’s test (****p* < 0.001, ***p* < 0.01, **p* < 0.05, and ns for *p* > 0.05).


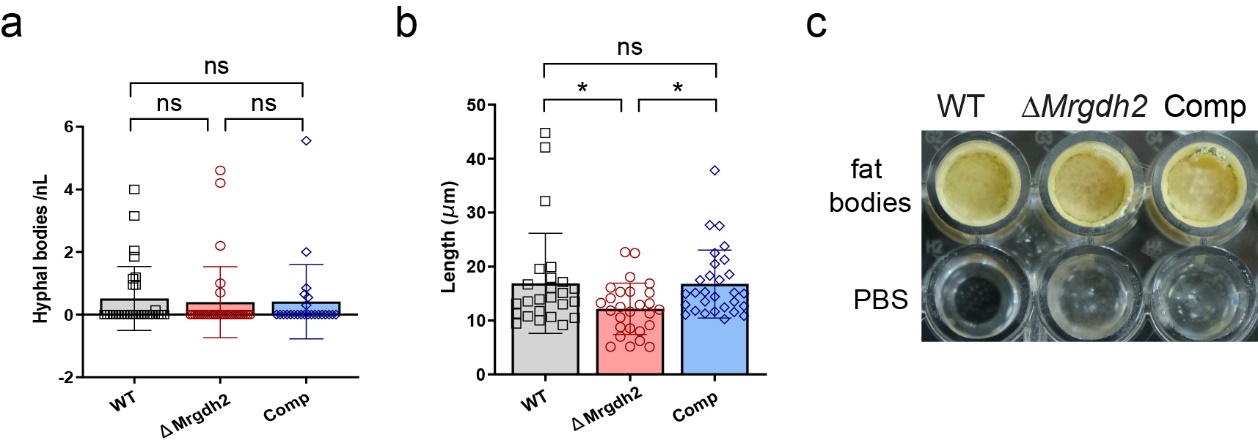


**Figure S8.** Phenotypes of fungal strains grown in hemolymph or medium supplemented with insect fat bodies. (a) The number of hyphal bodies in the hemolymph of *S. litura* larvae injected with the WT, Δ*Mrgdh2* or Comp strain for 48 h. (b) The length of hyphal bodies in the hemolymph of *B. mori* larvae injected with the WT, Δ*Mrgdh2* or Comp strain for 48 h. (c) The WT, Δ*Mrgdh2* and Comp strains were cultivated on PBS supplemented with fat bodies from *G. mellonella* larvae for 7 days. Values are means ± SD of replicates, the comparisons were performed by one-way ANOVA with Turkey’s test (**p* < 0.05, and ns for *p* > 0.05).

**
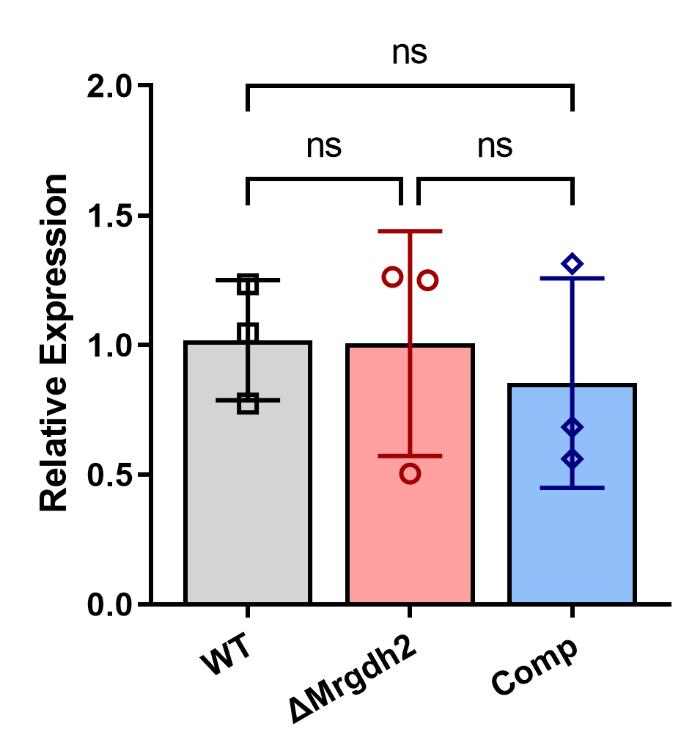
**

**Figure S9.** Relative expressions of *MrPacC* in appressoria. The WT, Δ*Mrgdh2* and Comp strains were cultivated on cicada wings for 48 h to induce appressoria. The expression levels of *MrPacC* (*MAA_06038*) genes were analyzed by qRT-PCR and beta-actin was used as a reference gene. Values represent means ± SD of replicates. No significant difference (ns, *p* > 0.05) was observed between groups based on one-way ANOVA with Turkey’s test.

**Table S1** Primers used in this study.

| Primer | Sequence (5’ 🡪 3’) |
| --- | --- |
| GDH2-UF | CGGGATCCTCAGAGGGCGTGAAATTCCT |
| GDH2-UR | CGGGATCCAGGACAATAGACGCCCCAAA |
| GDH2-DF | CACTAGTATGTCAGTGGACCTAGATACGA |
| GDH2-DR | CACTAGTCAAGCGGAAGCTAAGAGC |
| GDH2-Comp-F | GCTCTAGATGTACTTGCCACAGGTTTGCTG |
| GDH2-Comp-R | GCTCTAGAATCCCTCGGATTTTCGTACGT |
| GDH2-Test-F | TGCTTATGGCCCAGTTTCGT |
| GDH2-Test-R | CCAGCACGTCAGATAGGGAC |
| GDH2-RT-F | AAGCCTTCTCCCCAACCTAC |
| GDH2-RT-R | TTCCATGTCCAGGCGAATCT |
| Tubulin-F | CCCCGGTCAGTTGAACTCTG |
| Tubulin-R | GAAGATGGCAGAGCAGGTCA |
| EF-1-qF | CGACATTGCCCTCTGGAAGT |
| EF-1-qR | CCAGTACCGGCAGCGATAAT |
| Actin-qF | AATCTGGCGGTATCCACGTC |
| Actin-qR | CTGCATACGGTCGGAGAGAC |
| MrPacC-qF | GCCAAGCCCCCAAGCTACTA |
| MrPacC-qR | CATAGCCGCCAGCAGAAGTA |
