# Supplementary figures and images for: Glutamate dehydrogenase 2 is required for virulence by facilitating fungal growth in the host hemocoel
Source: Virulence. 2025 Nov 26;16(1):2591402. doi: 10.1080/21505594.2025.2591402 (PMC12667647; doi:10.1080/21505594.2025.2591402)

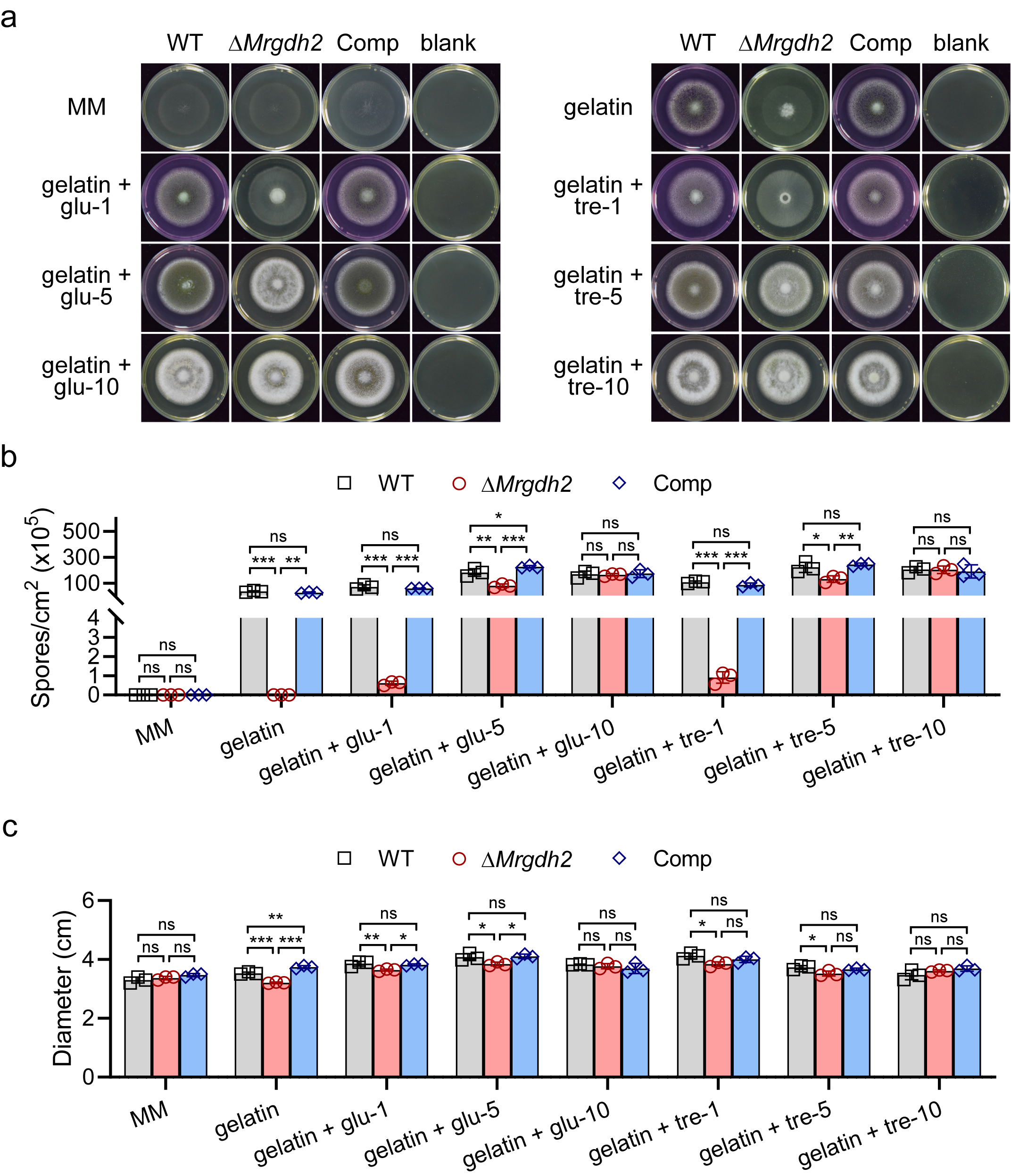

Supplement: Figure S6.tif [file KVIR_A_2591402_SM4621.tif]

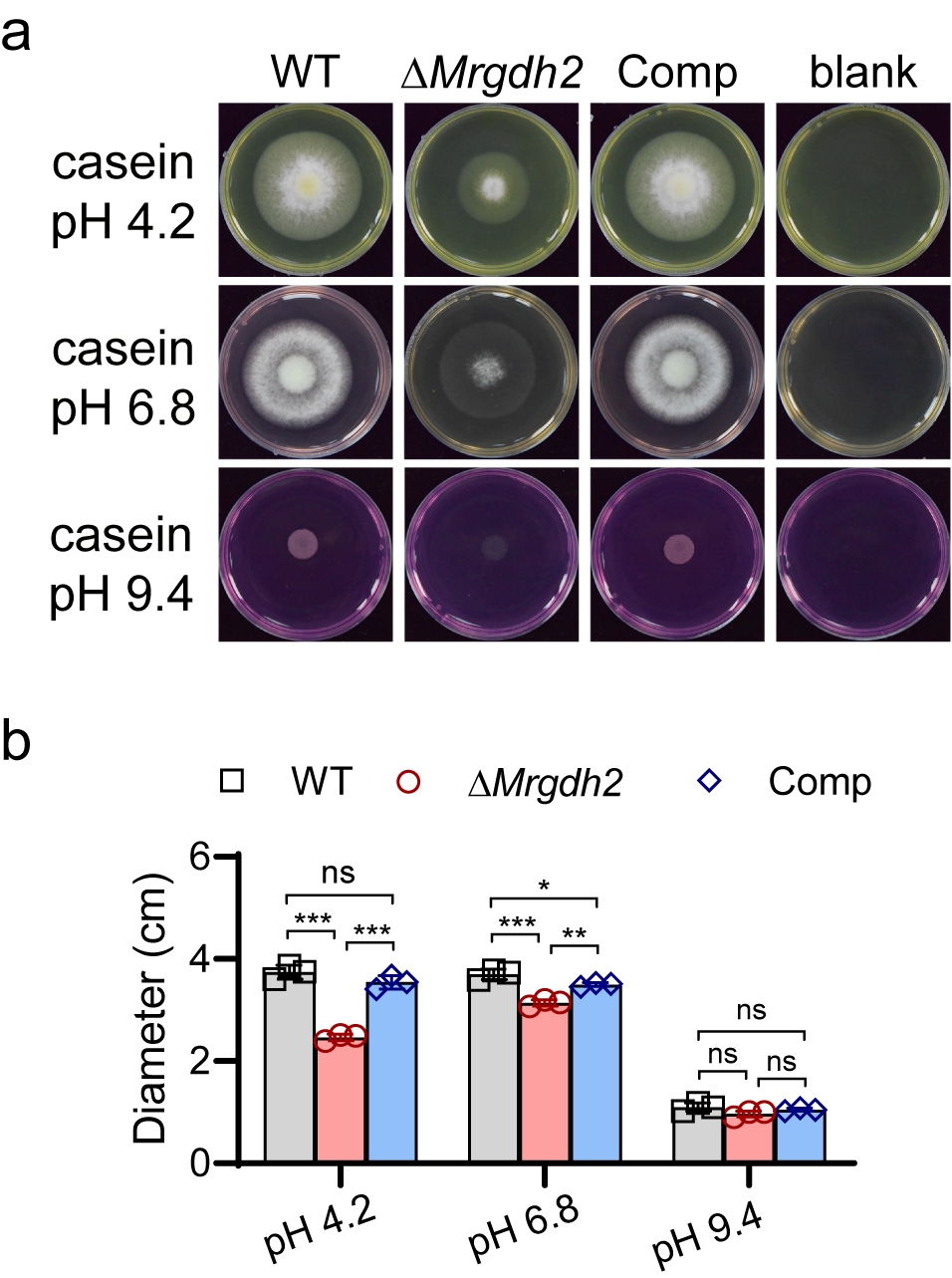

Supplement: Figure S7.tif [file KVIR_A_2591402_SM4620.tif]

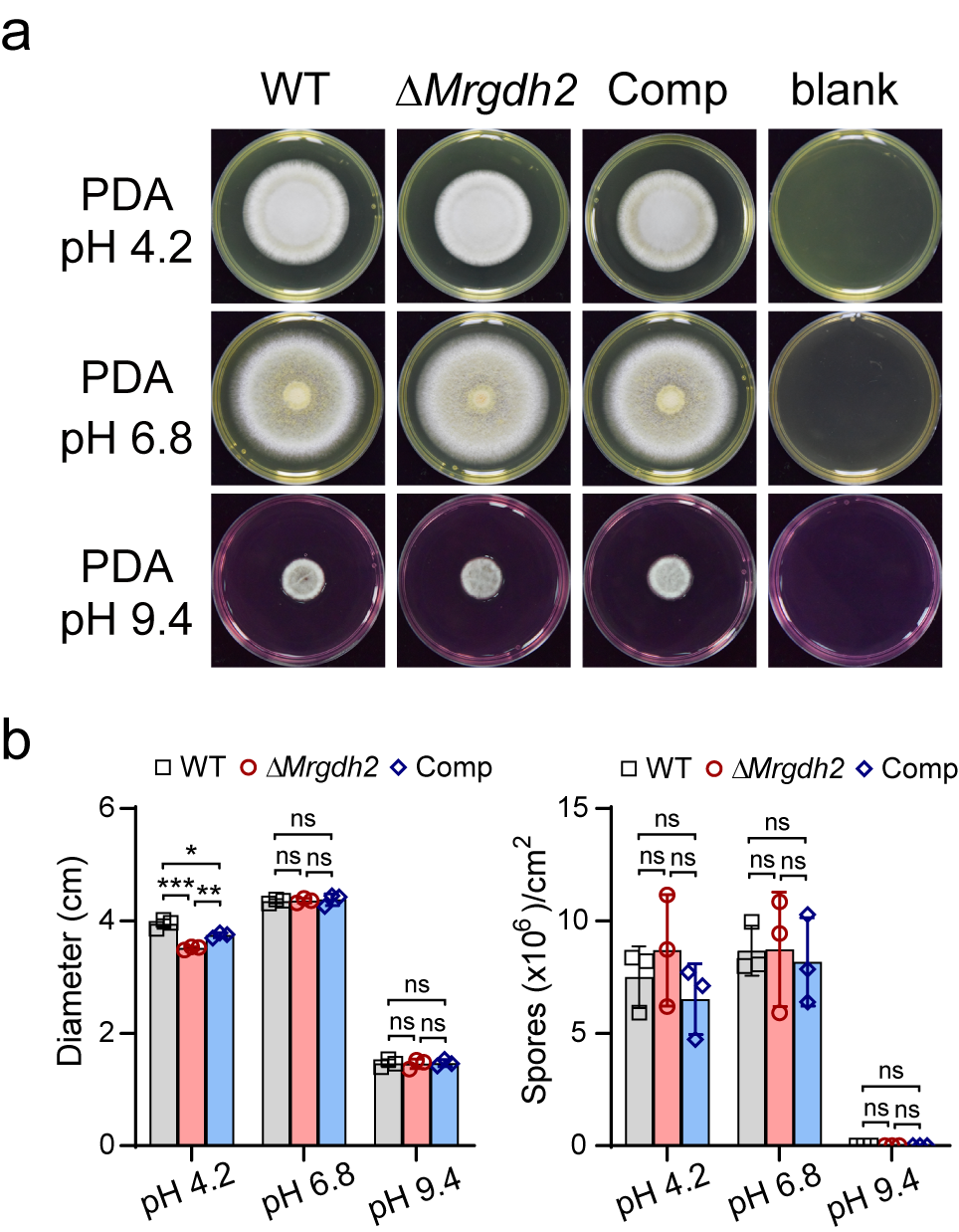

Supplement: Figure S4.tif [file KVIR_A_2591402_SM4619.tif]
